# Acute myocardial infarction in a patient positive for lupus anticoagulant: a case report

**DOI:** 10.1186/s12872-019-1153-9

**Published:** 2019-07-12

**Authors:** Kota Murai, Kenji Sakata, Tadatsugu Gamou, Yoji Nagata, Hayato Tada, Masaya Shimojima, Hirofumi Okada, Kenshi Hayashi, Masa-aki Kawashiri

**Affiliations:** Department of Cardiovascular and Internal Medicine, Kanazawa University Graduate School of Medicine, 13-1 Takara-machi, Kanazawa, 920-8640 Japan

**Keywords:** Acute myocardial infarction, Antiphospholipid syndrome, Systemic lupus erythematosus, Atherosclerosis

## Abstract

**Background:**

Autoimmune diseases, such as systemic lupus erythematosus (SLE), are associated with thrombosis and atherosclerosis. Presence of lupus anticoagulant is an independent risk factor for atherosclerotic diseases.

**Case presentation:**

A 56-year-old man with past history of hypertension, and cerebral infarction was admitted to our hospital owing to acute chest pain. He was diagnosed with acute myocardial infarction based on his symptoms and electrocardiogram results, which demonstrated ST elevation in the precordial leads. Coronary angiography images revealed total occlusion at the proximal site of the left anterior descending artery. A drug-eluting stent was deployed, which successfully recovered coronary blood flow. The patient had fever of unknown cause when he was 30 years old; on admission, he presented with a low-grade fever and reddish exanthema affecting both cheeks. Based on his physical signs as well as elevated antinuclear antibodies (anti-double-stranded DNA), decreased lymphocytes, and a positive direct Coombs test, he was diagnosed with SLE. Owing to a positive lupus anticoagulant test, he was also suspected to have antiphospholipid syndrome (APS). Triple antithrombotic therapy, including dual antiplatelet therapy with aspirin and clopidogrel during coronary stenting and single anticoagulation therapy with warfarin, was initiated.

**Conclusions:**

Careful diagnosis of autoimmune diseases should be performed in patients with thrombosis and atherosclerosis. Moreover, risk factors for coronary artery disease should be strictly controlled in patients with APS.

## Background

Antiphospholipid syndrome (APS) is an important cause of acquired thrombophilia and recurrent miscarriages [1]. Venous and arterial thromboses are the common symptoms of APS; however, APS reportedly causes atherosclerotic cardiovascular diseases [2, 3]. Here, we report a case of acute myocardial infarction caused by coronary artery stenosis and thrombosis with lupus anticoagulant.

## Case presentation

A 56-year-old Japanese man was admitted to our hospital owing to complaints of acute chest pain. At the age of 30 years, he had fever and hypersensitivity to sunlight; the causes of which were undetermined. He was diagnosed with hypertension during his 30s and was treated with antihypertensive drugs. Despite having well-controlled blood pressure levels, he experienced cerebral infarction at the age of 54 years; subsequently, antiplatelet therapy was initiated with 75 mg/day of clopidogrel.

**Fig. 1:**
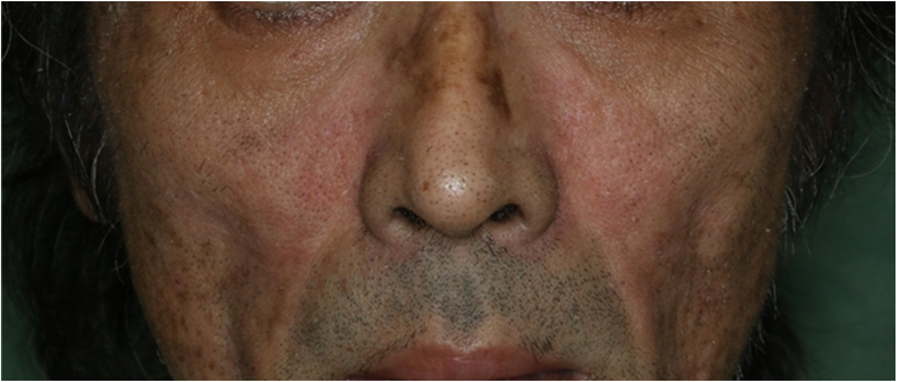
Facial appearance of the patient. Reddish exanthema was observed on both cheeks

**Fig. 2:**
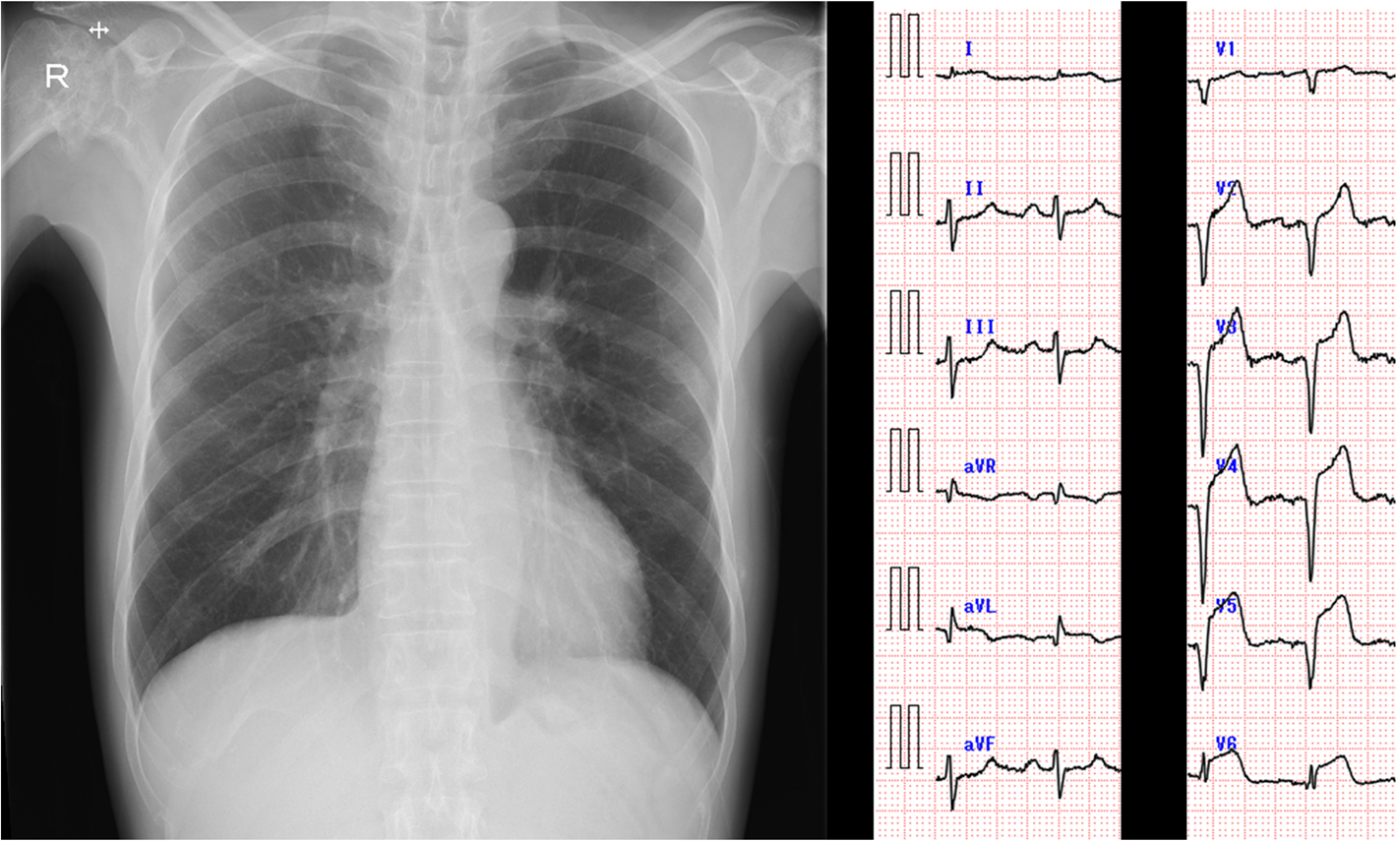
Chest X-ray and electrocardiogram results. The chest X-ray results were normal. The electrocardiogram results revealed ST elevation and a QS pattern in the precordial leads

On admission, the patient’s blood pressure level and heart rate were 126/70 mmHg and 80 bpm, respectively, and he had reddish exanthema on both cheeks (Fig. 1). His physical examination did not reveal any other abnormal findings. Although chest X-ray images did not reveal any significant finding, electrocardiograms exhibited prominent ST elevation in the precordial leads, thereby suggesting acute anteroseptal myocardial infarction (Fig. 2). Results of laboratory analyses revealed elevated levels of cardiac enzymes, such as creatine kinase (1511 IU/L), troponin T (1.400 ng/mL), and lactate dehydrogenase (454 IU/L). Conversely, cardiovascular risk factors, such as total cholesterol (162 mg/dL), low-density lipoprotein cholesterol (95 mg/dL), and hemoglobin A1c (6.5%), were desirable for the primary prevention of coronary heart disease. The patient had no history of diabetes mellitus or dyslipidemia; further, he was a current smoker, although the frequency was low (two cigarettes per day for the past 30 years), and was underweight (body mass index, 17.9 kg/m^2^).

**Fig. 3:**
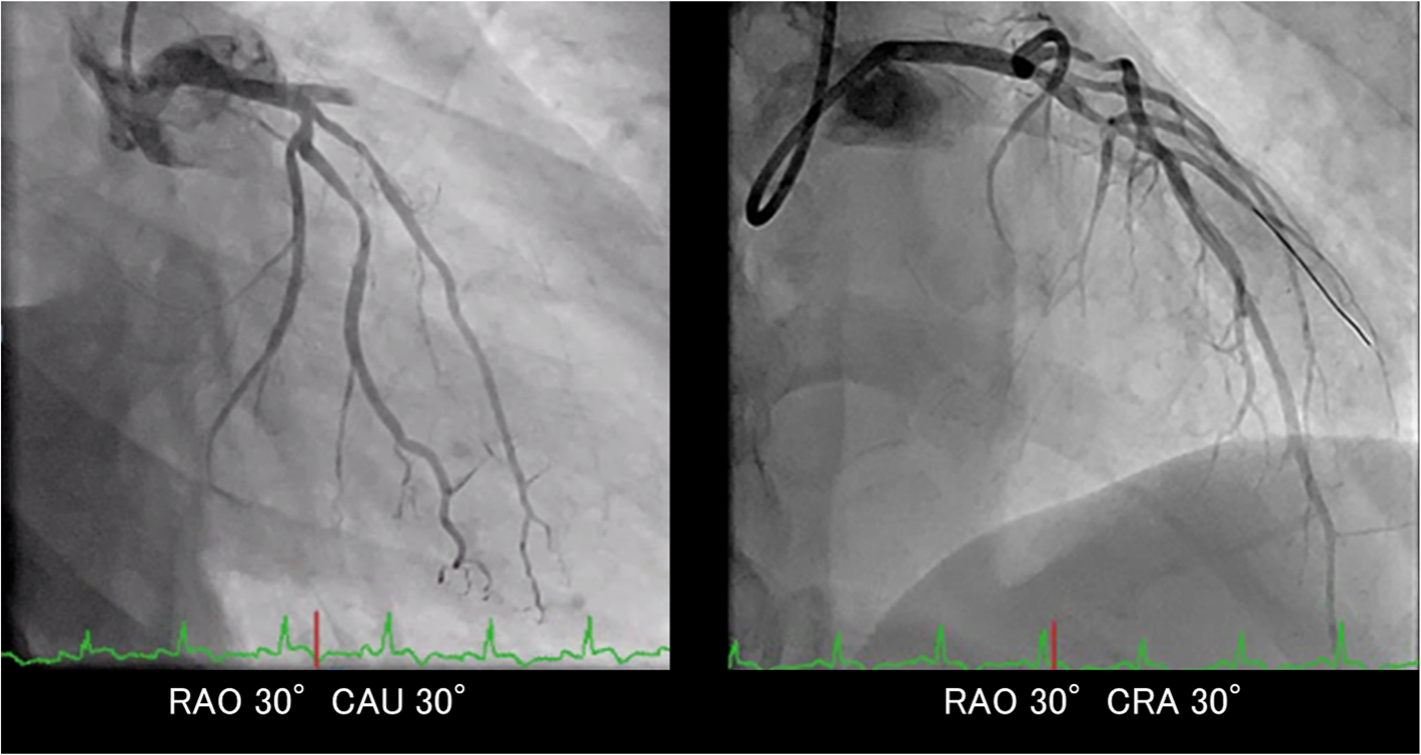
Coronary angiogram of the left coronary artery. Left panel: Total occlusion was observed at the proximal lesion of the left anterior descending artery. Right panel: Access using a guide wire resulted in partial recanalization of the occluded site

**Fig. 4:**
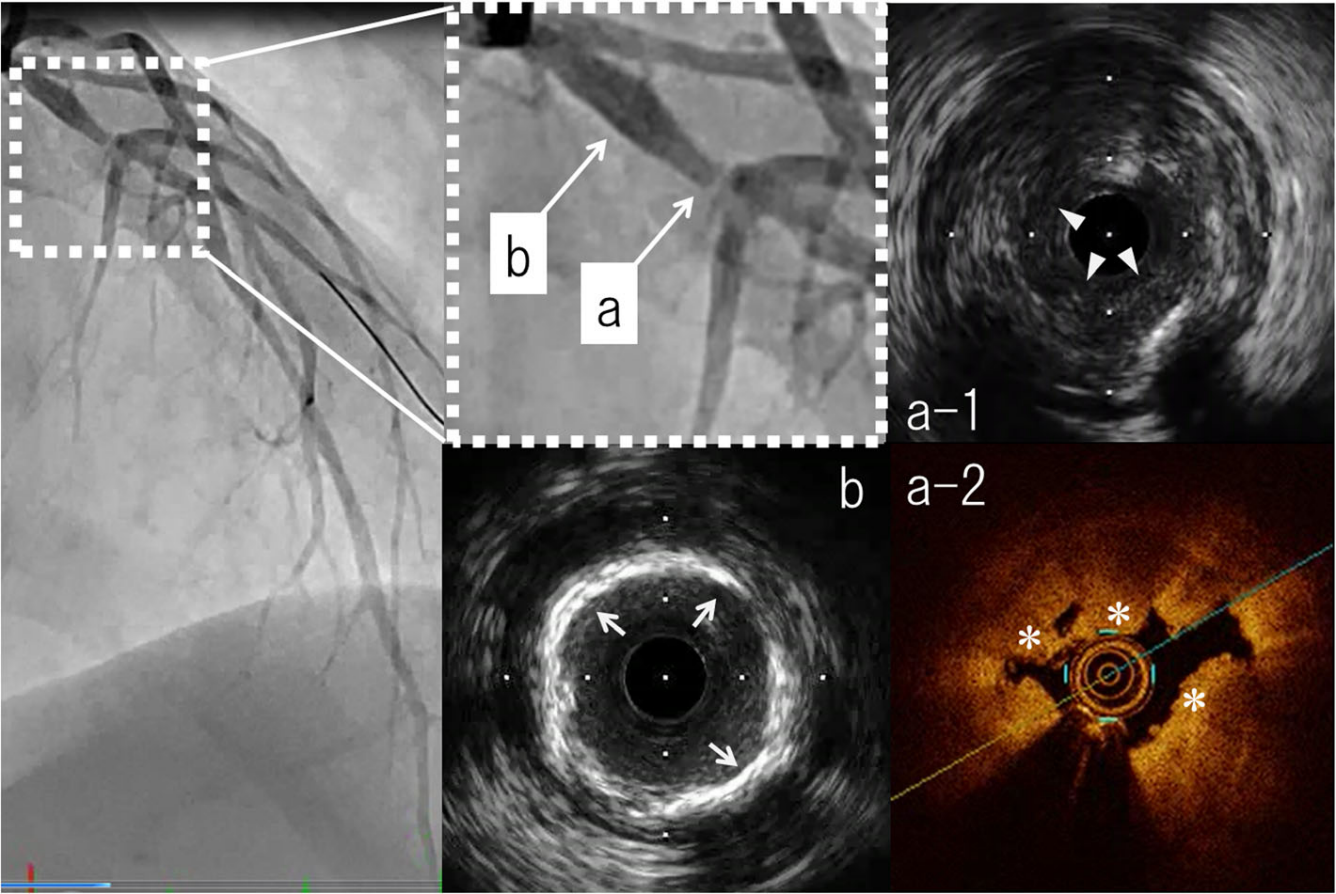
Intracoronary images using intravascular ultrasound (IVUS) and optical coherent tomography (OCT). **a-**1 At the occlusive site, intravascular ultrasound (IVUS) demonstrated a low-intensity structure with an irregular surface (arrow heads), suggesting the presence of a thrombus **a-**2 Optical coherent tomography (OCT) revealed that the thrombus showed strong signal attenuation and obscured underlying vascular structures (asterisk), suggesting the presence of a red thrombus, mostly consisting of red blood cells. **b** Proximal to the occlusive site, IVUS demonstrated a high intensity of the luminal surface and signal attenuation (arrows), indicating the presence of atherosclerotic plaque with calcification

Results of emergency coronary angiography revealed abrupt and total occlusion of the left anterior descending artery (LAD) (Fig. 3, left). Access using a guide wire resulted in partial recanalization of the occluded site (Fig. 3, right). The morphology of the lumen and vessel wall was observed by performing intravascular ultrasound (IVUS) and optical coherence tomography (OCT). IVUS images revealed an extremely large thrombus at the occlusive site (Fig. 4a-1) and an atherosclerotic plaque with calcification proximal to the occlusive site (Fig. 4b). OCT images revealed that the thrombus exhibited strong signal attenuation and obscured underlying vascular structures, suggesting that it was a red thrombus mostly comprising red blood cells (Fig. 4a-2). Interestingly, there was no distinct evidence of a ruptured plaque. Following balloon dilatation, a drug-eluting stent was successfully deployed, which completely recovered coronary blood flow in LAD.

The maximum creatine kinase level that contributed to apical cardiac aneurysm formation was 6381 IU/L. On hospital day 9, anticoagulation therapy with warfarin was initiated to prevent the formation of a left ventricular thrombus. Accordingly, triple antithrombotic therapy was initiated, which included dual antiplatelet therapy with aspirin and clopidogrel during coronary stenting and single anticoagulation therapy with warfarin.

Interestingly, the patient had a low-grade fever since admission but no evidence of infection. Based on the rashes on his cheeks, coexistence of a connective tissue disease was suspected. Additional laboratory tests demonstrated elevated levels of antinuclear antibodies (titer, 1/1280) and anti–double-stranded DNA antibodies (22 IU/mL). Furthermore, the lymphocyte count was low (550 cells/μL) and direct Coombs test result was positive. Based on these findings, the patient was diagnosed with systemic lupus erythematosus (SLE). In addition, the patient was positive for lupus anticoagulant, although a definitive diagnosis of APS requires consistently positive antiphospholipid antibody test results after > 12 weeks. Triple antithrombotic therapy was decided to be continued for as long as possible. The patient was discharged on hospital day 26.

## Discussion and conclusions

Atherosclerosis and systemic thrombosis may occur as a result of various risk factors, including hypertension, diabetes mellitus, dyslipidemia, and smoking; in addition, autoimmune diseases have been associated with the development of atherosclerotic diseases and systemic thrombosis [4]. Our patient had a history of hypertension and smoking and experienced myocardial infarction, and this could be regarded as a typical clinical course. However, he also exhibited clinical signs of SLE; therefore, we conducted tests to detect lupus anticoagulant, anticardiolipin antibody, and anti-CLGPI, which are the criteria for APS. Ando et al. reported that 0.31 and 0.37% of patients with ST-elevated and non-ST-elevated myocardial infarction, respectively, had SLE [5]. Conversely, Pons-Estel et al. reported that approximately 40% of patients with SLE had antiphospholipid antibodies and that 50–70% of them may develop APS after 20 years of follow-up [6]. González-Pacheco et al. reported a similar case of a healthy 28-year-old man who showed no sign of coronary risk factors but had APS and experienced acute left main coronary artery thrombosis and ischemic stroke [7]. These studies may include coronary artery thrombosis and atherosclerotic myocardial infarction owing to the progression of coronary plaque. In the current case, multiple intravascular imaging modalities revealed thrombi with severe atherosclerosis in the coronary artery.

Our patient required triple antithrombotic therapy, including dual antiplatelet therapy during coronary stenting and single anticoagulation therapy for preventing left ventricle thrombosis; he also required secondary thromboprophylaxis for APS. Triple antithrombotic therapy is associated with a high risk of bleeding [8]. Despite many receiving anticoagulation therapy, the recurrence rate of thrombosis in patients with APS is very high (2–10% per year) [9].

APS is associated with both thrombosis and atherosclerosis [2, 3]. A large amount of atherosclerotic plaques were observed via positive vascular remodeling by performing IVUS and OCT, despite the presence of well-controlled classical coronary risk factors in our patient. Careful diagnosis of co-morbid APS should be performed in patients with thrombosis and atherosclerosis, particularly in those showing specific symptoms associated with SLE, such as rash or arthritis, with the absence of classical risk factors for cardiovascular diseases. In addition, we strongly suggest that cardiovascular risk factors should be strictly controlled in patients with APS.

## Data Availability

All data generated or analyzed during this study are included in this published article.

## Abbreviations

APS: Antiphospholipid syndrome
IVUS: Intravascular ultrasound
LAD: Left anterior descending artery
OCT: Optical coherence tomography
SLE: Systemic lupus erythematosus

## References

[1] Hughes GR (1993). The antiphospholipid syndrome: ten years on. Lancet..

[2] Mineo C (2013). Inhibition of nitric oxide and antiphospholipid antibody-mediated thrombosis. Curr Rheumatol Rep.

[3] Soltész P, Szekanecz Z, Kiss E, Shoenfeld Y (2007). Cardiac manifestations in antiphospholipid syndrome. Autoimmun Rev.

[4] Corban MT, Duarte-Garcia A, McBane RD, Matteson EL, Lerman LO, Lerman A (2017). Antiphospholipid syndrome: role of vascular endothelial cells and implications for risk stratification and targeted therapeutics. J Am Coll Cardiol.

[5] Ando T, Adegbala O, Akintoye E, Ashraf S, Briasoulis A, Takagi H (2019). Acute myocardial infarction outcomes in systemic lupus erythematosus (from the nationwide inpatient sample). Am J Cardiol.

[6] Pons-Estel GJ, Andreoli L, Scanzi F, Cervera R, Tincani A (2017). The antiphospholipid syndrome in patients with systemic lupus erythematosus. J Autoimmun.

[7] González-Pacheco Héctor, Eid-Lidt Guering, Piña-Reyna Yigal, Amezcua-Guerra Luis M., Aldana-Sepúlveda Natalia, Martínez-Sánchez Carlos (2014). Acute left main coronary artery thrombosis as the first manifestation of systemic lupus erythematosus and catastrophic antiphospholipid syndrome. The American Journal of Emergency Medicine.

[8] Cavallari I, Patti G (2018). Meta-analysis comparing the safety and efficacy of dual versus triple antithrombotic therapy in patients with atrial fibrillation undergoing percutaneous coronary intervention. Am J Cardiol.

[9] Bazzan M, Vaccarino A, Stella S, Bertero MT, Carignola R, Montaruli B (2013). Thrombotic recurrences and bleeding events in APS vascular patients: a review from the literature and a comparison with the APS Piedmont cohort. Autoimmun Rev.

